# A Lightweight and Affordable Wearable Haptic Controller for Robot-Assisted Microsurgery

**DOI:** 10.3390/s24092676

**Published:** 2024-04-23

**Authors:** Xiaoqing Guo, Finn McFall, Peiyang Jiang, Jindong Liu, Nathan Lepora, Dandan Zhang

**Affiliations:** 1 Department of Engineering Mathematics, University of Bristol, Bristol BS8 1QU, UK; 2 Hamlyn Centre for Robotic Surgery, Imperial College London, London SW7 2AZ, UK; 3 Department of Bioengineering, Imperial College London, London SW7 2AZ, UK

**Keywords:** robot-assisted microsurgery, teleoperation, haptic feedback

## Abstract

In robot-assisted microsurgery (RAMS), surgeons often face the challenge of operating with minimal feedback, particularly lacking in haptic feedback. However, most traditional desktop haptic devices have restricted operational areas and limited dexterity. This report describes a novel, lightweight, and low-budget wearable haptic controller for teleoperated microsurgical robotic systems. We designed a wearable haptic interface entirely made using off-the-shelf material-PolyJet Photopolymer, fabricated using liquid and solid hybrid 3D co-printing technology. This interface was designed to resemble human soft tissues and can be wrapped around the fingertips, offering direct contact feedback to the operator. We also demonstrated that the device can be easily integrated with our motion tracking system for remote microsurgery. Two motion tracking methods, marker-based and marker-less, were compared in trajectory-tracking experiments at different depths to find the most effective motion tracking method for our RAMS system. The results indicate that within the 4 to 8 cm tracking range, the marker-based method achieved exceptional detection rates. Furthermore, the performance of three fusion algorithms was compared to establish the unscented Kalman filter as the most accurate and reliable. The effectiveness of the wearable haptic controller was evaluated through user studies focusing on the usefulness of haptic feedback. The results revealed that haptic feedback significantly enhances depth perception for operators during teleoperated RAMS.

## 1. Introduction

The significance of haptic feedback is particularly highlighted in RAMS. Studies have demonstrated that the absence of haptic feedback in teleoperational systems can lead to longer operation durations and an increased propensity for errors [1]. Physiological tremors and involuntary motions caused by surgeons can reduce surgical accuracy and influence safety during microsurgical operations [2]. Robot-assisted microsurgery (RAMS) can overcome these limitations by incorporating advanced technologies in robotics and teleoperation [3], resulting in improved dexterity and precision [4]. Surgeons can teleoperate microsurgical robots based on real-time visual feedback [5]. While visual cues in RAMS are crucial for providing feedback to operators, they may not always be sufficient during complex and delicate procedures. The significance of haptic feedback is particularly highlighted in RAMS [6]. Studies have demonstrated that the absence of haptic feedback in teleoperational systems can lead to longer operation durations and an increased propensity for errors [7]. The development of a controller for teleoperation with haptic feedback that can be integrated into existing surgical robotic systems represents an active and ongoing trend.

Efforts have been made to integrate desktop haptic devices into existing surgical platforms to provide haptic feedback to surgeons. Studies have targeted the integration of haptic devices like Sigma.7 [8], SynTouch BioTac [9], etc., into da Vinci systems, as a solution to provide haptic feedback to operators. Similar efforts have been made to integrate haptic controllers (PHANTOM Omni, SensAble Technologies, Wilmington, MA, USA) [10] into another typical surgical platform, Raven. However, desktop haptic feedback devices are limited in their workspace and dexterity. Recently, the focus of teleoperation in RAMS has shifted towards developing remote controllers that provide haptic feedback on the fingertips of users, which are not only smaller and lighter but also more ergonomically designed [11]. Such advancements ensure operators can retain their natural motion with a flexible workspace during teleoperation.

Some of these devices achieve normal indentation on the fingertip through a dynamic platform, enhancing the user’s sensory experience in virtual environments [12,13]. Others focus on providing lateral skin stretch or relative tangential motion, creating a more realistic touch sensation [14,15,16]. These devices usually apply forces to fingertips via stiff surfaces, meaning softness cannot be effectively achieved.

Flexible structures (polymer films or fabrics), as well as soft materials (elastomers), are being explored for soft tactile displays. The former are driven by motors and rely on complex mechanical transmissions for deformation, resulting in intricate, cumbersome, and heavy mechanisms [16,17]. Soft materials (elastomers) are being investigated as a means to overcome this drawback, utilizing three techniques: dielectric elastomer actuators (DEAs) [18,19,20], electrostatic actuators [21], and pneumatic actuators [22,23]. Compared to dielectric elastomer actuators (DEAs) and electrostatic actuators, pneumatic actuators can achieve electrically safe, compact, and lightweight interfaces because the source used to drive the actuators is generated far from the point of actuation, allowing many actuators to be packed tightly together. However, these devices often necessitate complex manufacturing techniques that are not only time-consuming but also costly, particularly when adapting the designs to accommodate different shapes or user-specific requirements. Furthermore, the integration of these devices into existing surgical platforms also presents challenges.

A promising strategy emerges from the integration of wearable haptic devices and motion tracking technology. Recent innovations include motion tracking exoskeleton-based controllers equipped with force feedback. An example of this technology is the HaptX Glove, which offers haptic feedback in teleoperated tasks [24]. Despite its capabilities, the HaptX Glove’s heavy weight and high cost impede its widespread application, particularly in RAMS.

Here, we design and prototype a lightweight wearable glove-like haptic device equipped with a novel soft haptic interface to provide haptic feedback on the fingertips, along with multiple sensing modalities for motion tracking. We also investigate advanced multi-material additive manufacturing technologies to enable the fast prototyping of customized haptic interfaces, which can be easily integrated into wearable haptic controllers of different sizes and shapes. The main contributions of this paper are listed as follows.

(i) Controller-integrated Haptic Feedback and Motion Tracking: We designed a wearable glove-like haptic controller for RAMS. The controller integrates both haptic feedback and motion tracking systems. Haptic feedback is provided by soft haptic interfaces wrapped around the user’s fingertips, while a multi-sensor fusion-based method is used for motion tracking, translating the surgeon’s hand gestures into control commands for the micro-surgical robot. This device aims to provide surgeons with a more natural and intuitive method to control the remote surgical robot.

(ii) Investigation of an Advanced Multi-Material Additive Manufacturing Technique for the Fabrication of a Haptic Interface: We investigated a novel liquid and solid hybrid 3D co-printing technology to fabricate a pneumatically driven haptic interface, which is integrated into the wearable controller to provide haptic feedback on the user’s fingertips. This technology opens up new possibilities for customizing and fast prototyping haptic interfaces to various types of wearable controllers.

(iii) Experimental Assessments of Wearable Haptic Controllers: We designed trajectory tracking experiments to compare marker-based and markerless motion tracking methods, aiming to identify the most effective approach for our RAMS and determine the optimal tracking range. Simultaneously, the experiments compared three fusion algorithms across different planes to confirm that the UKF was the most accurate and reliable for our RAMS. Finally, we conducted user studies to assess the effectiveness of the haptic feedback system in enhancing users’ depth perception during teleoperated microsurgical training.

## 2. Related Work

### 2.1. Grounded Haptic Device in Teleoperated RAMS

Most teleoperated microsurgical robots employ grounded (desktop) haptic devices to implement teleoperation for RAMS. Touch (3D Systems, Rock Hill, SC, USA) and Omega (Force Dimension, Nyon, Switzerland) are widely used commercial grounded haptic devices, which have been used for different surgical tasks, including dental restorations and medical simulation training [25]. Quek et al. [26] developed a haptic device that could be attached to the end-effector of the Omega device (Force Dimension). Han et al. [27] presented a similar device designed to assist surgeons during magnetic-resonance-guided biopsy procedures. These types of haptic devices are very accurate and able to provide a wide range of forces. However, compared to wearable devices, grounded haptic devices have significant disadvantages in terms of their large size and weight. In addition, the workspace of grounded haptic devices is limited due to their size and/or functional features.

### 2.2. Wearable Haptic Device in RAMS

In contrast, wearable devices are lighter, impose fewer mobility restrictions, and offer a larger workspace. Glove-based haptic exoskeletons currently available on the market include Dexmo (Dextra Robotics, Shenzhen, China) and CyberGrasp (CyberGlove Systems LLC, San Jose, CA, USA) [28]. These devices have been employed in virtual environments to provide users with an immersive sense of control, delivering real-time feedback by simulating resistance for a realistic gripping sensation. However, the complexity of mechanisms leads to the significantly high prices of these devices. Also, the weight of such devices is comparatively high, which may cause fatigue to operators during teleoperation [29]. On the other hand, finger-worn haptic devices primarily focus on providing tactile stimulation to the fingertips without relying on exoskeletons [30]. Presently, finger-worn haptic devices are developed using three main techniques: vibration, indentation, and lateral stretching [12,13,14,15,16].

While finger-worn haptic devices excel in generating force feedback, especially in shape rendering, they struggle to effectively achieve soft rendering due to their reliance on rigid surfaces for applying force to the fingertips [31,32]. To develop haptic devices with soft rendering capabilities, a strategy involves utilizing motor-driven flexible structures for deformation. Simone Fani developed a wearable fabric yielding display (W-FYD) [16], which used a layer of isotropic elastic fabric (Superbiflex HN by Mectex S.P.A) in the device as interaction surface for the user. Another softness-display device using a flexible sheet, which is a 1-mm-thick hyper-gel sheet, was introduced by Takahiro Endo for a muti-fingered robot [17]. However, these devices rely on intricate mechanical transmissions, typically leading to complex, cumbersome, and weighty mechanisms.

To address these limitations, another approach involves the use of soft materials, allowing for deformation without the need for complex mechanical transmissions. Three techniques being explored in this direction include dielectric elastomer actuators (DEAs), electrostatic actuators, and pneumatic actuators. The primary limitation for DEAs in haptic interfaces, as well as for purely electrostatic actuators, is the high current requirement for driving voltage [19]. In contrast, pneumatic actuation has emerged as an attractive option for activating haptic interfaces due to its swift response time and ability to generate substantial force, all while offering simplicity, electrical safety, and cost-effectiveness [22,23].

Several haptic displays have been manufactured using microelectromechanical systems (MEMS) technology. The latest research presents examples of combining pneumatic and electrostatic elements, including a novel deformable display featuring an embedded electroosmotic pump (EEOP) [33]. The integration of pneumatic and electrostatic components provides haptic interfaces with programmable soft surfaces, enabling them to have shape-rendering capabilities. However, their actuators rely on complex fabrication processes.

Federico Carpi described a pneumatically-driven fingertip display of softness. It consists of a small plastic chamber enclosed by a thin elastic membrane that can be pressurized with air to deform the membrane [34]. It was made of off-the-shelf materials and components, without a special manufacturing process. However, it encountered the challenge of accurately controlling the contact area and, consequently, regulating the softness that could be displayed.

## 3. Methodology

### 3.1. System Overview

**Figure 1:**
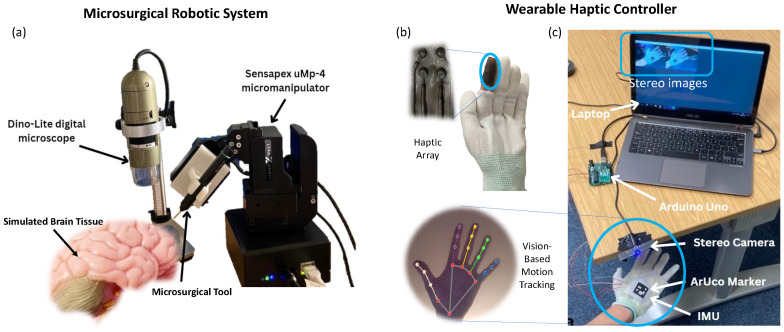
(**a**) Overview of the microsurgical robotic system. (**b**) Overview of the haptic interface. (**c**) Overview of multi-sensor fusion-based motion tracking for the wearable haptic controller.

The overview of the teleoperated RAMS system is shown in Figure 1a. The microsurgical robotic system is composed of a four-axis uMp-4 micromanipulator that can perform microsurgical tasks. A microsurgical tool (micro-needle) is mounted on the micromanipulator. A brain tissue model is used to simulate robot-assisted neurosurgery. A digital microscope is utilized to capture the lateral view of the operation scene, which can identify the distance between the tooltip and the simulated brain tissue. The captured images are subsequently processed using a deep learning-based object detection method based on You Only Look Once (YOLO) v5 architecture to estimate the force level for haptic feedback [35]. This information represents whether the surgical tool tip comes into contact with delicate tissues or not. Another digital microscope is used to capture a top-down operation view and provide visual feedback to operators. A PC is used to control the microsurgical robotic system via ethernet.

The wearable haptic controller is used to capture the hand motions to teleoperate the remote microsurgical robot, while a haptic interface is embedded into the glove to provide real-time haptic feedback. In pursuit of reliable motion tracking, an external camera and the embedded inertial measurement units (IMUs) on the glove-like wearable controller are used. An Arduino is used to process initial data obtained from IMUs, while the Arduino and camera are connected to a PC for multi-sensor fusion.

### 3.2. Motion Tracking

#### 3.2.1. Vision-Based Motion Tracking

For vision-based motion tracking, a low-cost stereo camera is used, which can acquire synchronized images from both lenses at a high frame rate of 60 fps. The stereo camera is mounted to a fixed position to capture the top-down view of the wearable controller. To ensure reliable tracking, marker-based detection is implemented by attaching an ArUco marker to the glove-like controller. The stereo camera can obtain the central point of the ArUco marker. After stereo calibration, the distance between the camera and the marker in the real-world coordinate can be obtained using the direct linear transformation (DLT) algorithm.

However, the marker-based method cannot enable the tracking of the finger motion to provide gripping information, and the method cannot perform well when the camera loses tracking of the marker. Therefore, a machine learning marker-less visual tracking method is implemented using an open-source library called MediaPipe (MP). The initial position of the hand is detected using a single-shot detector (SSD) model. MP uses an encoder–decoder feature extractor to reach an accuracy of 95.7% in palm detection. Following this, the hand landmark model, which is trained on over thirty thousand real-world images, performs precise keypoint localization of twenty-one 3D hand–knuckle coordinates inside the detected hand.

#### 3.2.2. Inertial Motion Tracking

An inertial measurement unit (IMU), which consists of three sensors: an accelerometer, a gyroscope, and a magnetometer, is assembled into the glove-like controller to provide supplementary information for motion tracking. An IMU is very useful when the camera undergoes fast motion. The *x*-axis on the IMU is aligned with the middle finger, as shown in Figure 1c. Initial sensor data processing is performed in Arduino before being integrated into the main system in Python using PySerial. To minimize integration errors when obtaining the velocity information through integrated acceleration value, calibration is applied to each sensor, ensuring that the measurement reads zero when the device is stationary. The calibration process involves obtaining a large number of readings, taking the mean, and subtracting this value from all future readings. A Madgwick filter is used for orientation estimation.

#### 3.2.3. Multi-Sensor Fusion

We use an extended Kalman filter (EKF) to fuse the pose estimation data provided by both the IMU and camera, which enables precise real-time motion tracking of the operator to generate control commands for robot control. An unscented Kalman filter (UKF) is applied for comparison. It utilizes a Gaussian distribution to represent the state distribution, characterized by a select group of sample points known as sigma points. These sigma points are processed through the actual nonlinear system rather than a linear approximation. Consequently, the state mean and covariance are accurately estimated up to the third order, a process achieved through the implementation of the unscented transform. This approach enhances the UKF’s capability to manage complex, nonlinear systems more effectively than the EKF.

### 3.3. Haptic Device

#### 3.3.1. Design

**Figure 2:**
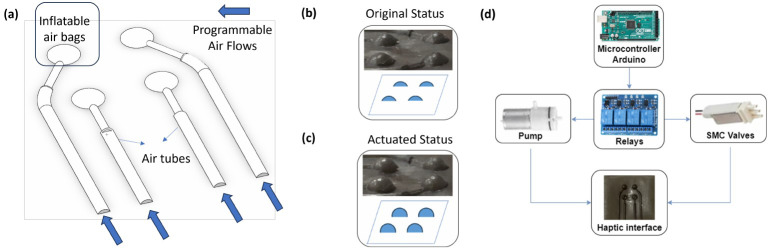
Haptic rendering device. (**a**) The structure and actuation principle. (**b**,**c**) An illustration of original and actuated status. (**d**) Overview of the control system for the haptic interface.

The haptic interface situated on the fingertip of the glove-like wearable controller is characterized as a pneumatically actuated haptic array. Each array in this setup comprises multiple inflatable airbags. Haptic feedback is relayed to the user by inflating these airbags with air. Both the quantity and the arrangement of the inflatable airbags are customizable to suit various specific applications. As a proof-of-concept, we have designed a haptic array consisting of four airbags arranged in a 2 × 2 grid pattern. The working principle of the haptic array is shown in Figure 2a. This design allows for the activation of different airbags in distinct locations, enabling the transmission of varied levels of haptic feedback in terms of intensity and pattern. Such feedback is crucial for conveying information about the force interactions between the microsurgical tooltip and the simulated tissue.

#### 3.3.2. Fabrication

In the realm of haptic feedback technology, conventional fingertip devices often suffer from significant drawbacks due to their bulkiness and the complexity involved in their construction. These devices typically rely on rigid materials and conventional manufacturing techniques that limit their design flexibility and user comfort, making them less suitable for applications requiring nuanced tactile feedback. Contrasting with these traditional methods, the haptic interface developed for our wearable controller utilizes an innovative liquid and solid hybrid 3D co-printing technology [36]. This approach employs liquid as the support material, as opposed to the traditional photo-curable soluble supports commonly used in additive manufacturing processes. This method not only streamlines the fabrication process of haptic arrays but also significantly reduces the labor and time involved in post-processing.

The core advantage of this novel printing technique lies in its ability to seamlessly integrate liquid and solid materials within a single fabrication cycle, thereby facilitating the direct creation of multi-material micro/meso fluidic channels. These channels play a crucial role in the operational efficacy of haptic devices, enabling the transmission of tactile feedback through fluidic mechanisms. Historically, the fabrication of such intricate arrays posed a considerable challenge, particularly in terms of removing support material from the delicate chambers without damaging the overall structure. However, with the advent of liquid support materials, these fluidic channels can now be cleared with unparalleled ease and precision. The process of purging the liquids from the fluidic channels is remarkably efficient, requiring only the application of a modest pressure differential. This can be readily achieved using a simple syringe, eliminating the need for complex or specialized equipment. Such an approach not only enhances the manufacturability of complex haptic arrays but also opens new avenues for the design and development of more sophisticated and user-friendly haptic feedback systems.

In enhancing the design and functionality of the haptic interface, Agilus 30 emerges as an exemplary printing material for crafting airbags and the interface’s remaining structure. Recognized for its exceptional tear resistance, Agilus 30 is a PolyJet photopolymer that exhibits remarkable durability against frequent flexing and bending. Its rubber-like properties not only mimic the appearance and texture of rubber-based products but also its functionality, positioning it as a quintessential choice for replicating human soft tissues. The adaptability of the haptic interface is further underscored by the customizable nature of its air chambers. Depending on the specific needs of the user and the task at hand, both the distribution and the number of air chambers within the interface can be tailored. This level of customization ensures that the device can deliver precise tactile feedback suited to various applications. Additionally, the interface’s softness can be finely adjusted by selecting the hardness level of the printing material used. This flexibility in material hardness allows for a more personalized and comfortable user experience, enhancing the overall efficacy and usability of the haptic device. Such detailed attention to material selection and structural design underlines the innovative approach taken in developing this haptic interface, ensuring that it not only meets but exceeds the requirements for delivering nuanced and realistic tactile feedback in diverse applications.

To identify the optimal parameters for the fabricated haptic interface, our investigation encompasses three distinct Shore A hardness levels: 30 A, 50 A, and 70 A. Through a series of comparative experiments involving airflow tests with durations of 0.5, 1, and 1.5 s, we discovered that the 50A hardness level was the most suitable. This preference for 50 A is attributed to its rapid recovery to its original shape after being inflated, demonstrating only minor variations in softness while showcasing an exceptional ability to withstand considerable pressure, notably outperforming the 30 A hardness level. Compared to the 70 A hardness level, the 50 A material significantly excelled in terms of flexibility and exhibited a more pronounced inflation effect, making it the superior choice for achieving precise and realistic tactile feedback. Furthermore, the research included an examination of haptic devices manufactured with different wall thicknesses, specifically 0.2 mm and 0.3 mm, to scrutinize their influence on the device’s performance. This aspect of the study aimed to explore how variations in thickness affect the device’s structural integrity, flexibility, and efficacy in conveying haptic sensations. The outcomes of these evaluations are expected to contribute valuable insights towards optimizing the design of the haptic interface, enhancing user engagement and comfort through more nuanced and lifelike feedback mechanisms.

Compared to its 0.3 mm counterpart (results can be found in Table 1), the 0.2 mm version (results can be found in Table 2) exhibited a significantly softer texture while still meeting the demands for high durability and quick shape recovery. Moreover, the internal volume of the 0.3 mm tactile device remained constant. Yet, in contrast to the 0.2 mm thickness, the outer spherical diameter of the cavity in the 0.3 mm device experienced an increase, leading to a reduced resolution of tactile feedback. Consequently, the 0.2 mm version, fabricated with a Shore A hardness of 50 A, was selected for the final prototype of the haptic interface, as illustrated in Figure 2b. This decision was based on the superior tactile sensation and feedback resolution offered by the 0.2 mm version, aligning with our objective to enhance the user experience through more nuanced and responsive haptic interaction.

#### 3.3.3. Actuation

The haptic interface is connected to solenoid valves (S070C, SMC Corporation, Tokyo, Japan), which are controlled by Arduino Mega2560 (Arduino, Somerville, MA, USA) through relays. A four-channel relay is employed to control solenoid valves. The pressure value of each chamber is controlled by adjusting the opening time of the valve. The actuation system employs DC mini air pumps operating at 12 volts as its pneumatic power source. When the airbags receive pressurized air, they inflate (see Figure 2c). The haptic interface is seamlessly integrated into the wearable controller. The signal processing for the soft haptic interface is shown in Figure 2d.

## 4. Experiments and Results Analysis

### 4.1. Evaluation of Motion Tracking

#### 4.1.1. Experiment Design and Evaluation Metrics

We employed a trajectory following task to evaluate the precision of the motion tracking algorithm. This task typically involves the participant following a predefined trajectory. This task simulates a basic microsurgical operation, i.e., navigating around sensitive tissues or maneuvering through narrow spaces. It is commonly utilized in training programs for surgeons to develop their microsurgical skills. Four metrics are used for quantitative analysis:

- Root-mean-squared error (RMSE): This metric quantifies the deviation between the actual motion path ${\vec{x}}_{k}$ and the predefined trajectory ${\hat{\vec{x}}}_{k}$, which can be calculated by $\mathrm{RMSE}=\sqrt{\frac{\sum_{k=1}^{n}{\left({\vec{x}}_{k}-{\hat{\vec{x}}}_{k}\right)}^{2}}{n}}$, where *n* is the number of points. RMSE offers a clear indicator of the tracking precision.
- Total path length *D*: Calculated by summing the end-effector’s trajectories throughout the task using Euclidean distances. This metric provides insight into the efficiency and smoothness of the motion.
- Tracking detection rate: To quantify the reliability of the motion tracking algorithms, we measured the detection rate $\gamma =\frac{n_{i}}{N}$, which represents the proportion of successfully detected frames $n_{i}$ out of the total frames *N*. This rate indicates the consistency of the tracking system.

#### 4.1.2. Results Analysis

We repeated the trajectory following task five times at three different depths *z* for each method. To evaluate the reliability of the motion tracking, we summarized the hand gesture detection rate. The results are summarised in Table 3, where ${\bar{n}}_{i}$, $\bar{N}$, and $\bar{\gamma }$ are the means of the detection count, total count, and detection rate.

It can be seen that the marker-based algorithm is more effective at shorter distances from the camera, while the marker-less algorithm outperforms the marker-based algorithm at greater distances. This is because the marker-less technique requires detecting the hand. When the hand is partly obscured at close distances, the stereo camera loses tracking. Conversely, the value of γ is lower for marker-based algorithms when they are further away because, as the markers become smaller, the camera resolution is too low to pick up the ArUco marker. The ArUco marker-based detection algorithm is used at depths of 8 > *z* > 4 cm due to its near-perfect detection rates of 0.97 and 0.99, respectively, within this range.

**Figure 3:**
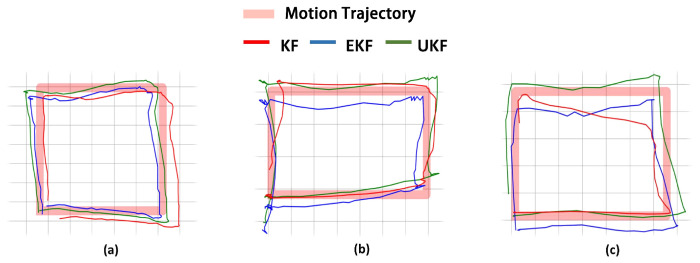
The performance of three types of filters across different planes. (**a**) Tracing in the *x*−*y* dimension. (**b**) Tracing in the *x*−*z* dimension. (**c**) Tracing in the *y*−*z* dimension.

We compare the performance of three types of filters when applied to motion tracking, as shown in Figure 3, including the Kalman filter (KF), extended KF (EKF), and unscented KF (UKF). We evaluate the trajectory following performance in different planes. The mean RMSE for the filters in each plane is shown in Table 4. These results indicate that the UKF consistently yields the lowest RMSE, outperforming the KF and EKF. This suggests that the UKF is the most effective algorithm, while the KF ranks as the least effective. In situations involving more complex, nonlinear movements, a decline in the KF’s effectiveness is anticipated, whereas the EKF and UKF are expected to sustain their accuracy levels.

### 4.2. Evaluation of Haptic Feedback

#### 4.2.1. Experiment Design and Evaluation Metrics

To assess the usefulness of the haptic interface, we conducted a user study involving six participants (two females and four males aged 25–30). Notably, three participants had prior experience with motion tracking-related games.

In our experiment, the soft tissue model is simplified as a regular sphere, and the biological needle is inserted perpendicular to the sphere’s centerline. YOLO v5 detects objects, including the midpoint of the biological needle and the center of the sphere representing the brain model, to estimate the movement distance of the biological needle relative to the center of the sphere, which corresponds to the insertion depth. Real-time depth information is presented numerically on the screen to provide visual feedback to the user. Additionally, it serves as a basis for estimating the tactile level.

**Figure 4:**
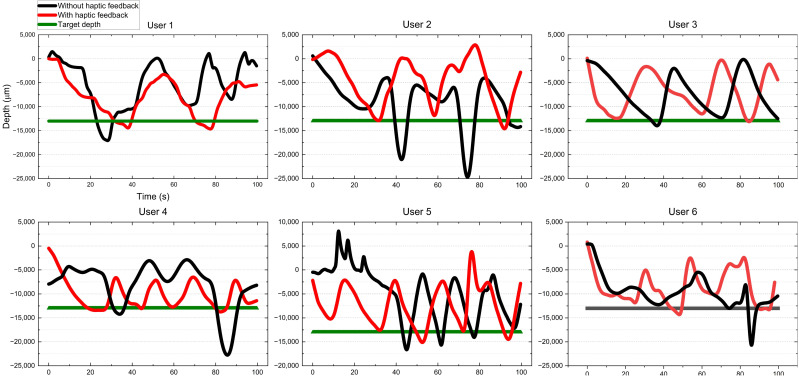
Depth of micro-needle insertion into tissue model. The graph depicts the depth trajectories of users operating a micro-needle inserted into a tissue model under conditions with and without haptic feedback. Each task involves inserting the micro-needle to the target depth and subsequently withdrawing it until it exits the tissue surface. Throughout the experiment, users repeat this process until the allocated time elapses. The local minima depicted on the graph mark the actual depth reached by the micro-needle, with their count indicating the number of tasks accomplished during the experimental period. This observation highlights the varying task completion rates among different users within the given experimental timeframe.

**Figure 5:**
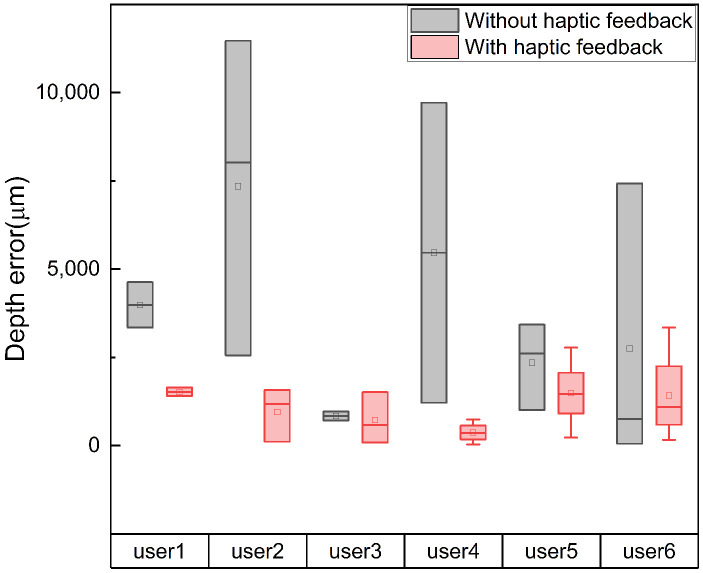
Depth errors. Depth error signifies the variance between the local depth minima in the trajectory and the predetermined target depth. The figure illustrates the error associated with each local minimum for every user, considering both scenarios, with and without haptic feedback. The error count differs among users, indicating variations in the number of tasks completed by each individual within the experimental timeframe.

**Figure 6:**
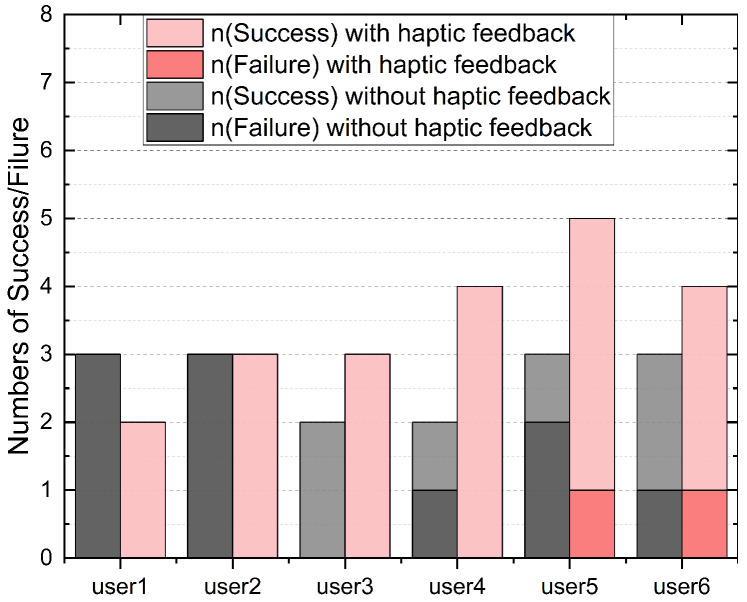
Success rate. Depth errors within an acceptable range are considered successful. This chart logs the overall task count completed by each user within the experimental duration, encompassing both successful and unsuccessful attempts, under scenarios with and without haptic feedback.

**Figure 7:**
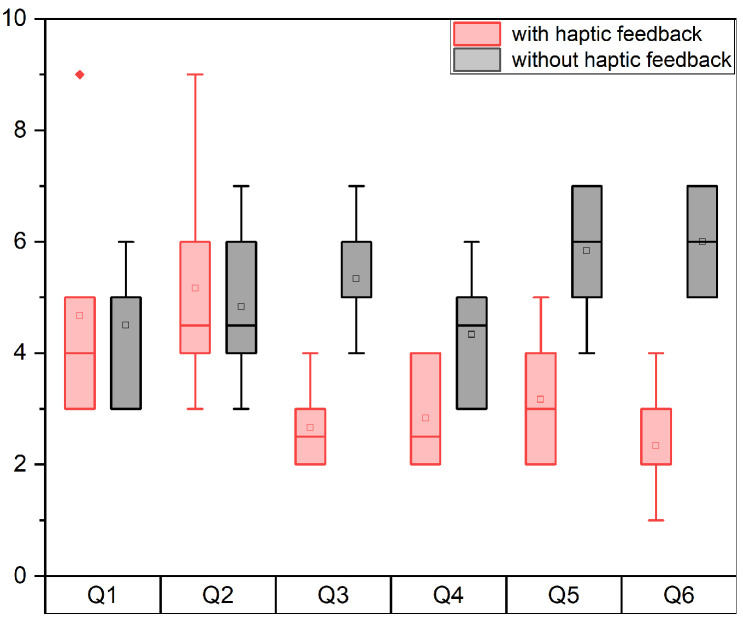
Result of the questionnaire with and without haptic feedback.

A needle insertion task was utilized in the user studies, requiring the participants to insert the tip of the micro-needle into a targeted area within a set time frame. The needle tip was pre-positioned at a distance of 0.8 cm from the soft tissue. The predetermined target insertion depth was 0.5 cm. A complete task cycle was defined as the participant inserting the micro-needle from the initial position into the soft tissue to the target depth and then withdrawing it from the tissue. Users repeated this task cycle iteratively until the task time expired. Our motion tracking system selected the optimal tracking plane to control the axial movement of the needle. As the optimal tracking range for the hand scale was 8>z>4 cm, the ratio of motion scales between the tracking system and the robot system was 2:1, to ensure that the needle remained within the optimal tracking range during the task cycle. Each participant performed the task six times in total. In three sets, participants received visual feedback from the screen and haptic feedback from the wearable controller. The other three sets involved solely visual feedback. The experiment recorded the robot’s kinematic data and observation data (distance between the robot’s end-effector and the brain tissue model during the tracking process). After completing six sets of trials, participants were asked to fill out the National Aeronautics and Space Administration Task Load Index (NASA-TLX) [37], a subjective workload assessment tool. The evaluation metrics were as follows:

- **Depth**: We measured the depth of micro-needle insertion into the tissue model to assess users’ perception of depth during the task. As illustrated in Figure 4, this displayed the trajectory of needle insertion under both haptic and non-haptic feedback conditions. The local minima in the depth trajectory correspond to the points where the participant believed they reached the target depth. Analyzing these minima revealed differences in users’ perception of target depth with and without haptic feedback.
- **Depth errors**: We quantified the difference between the actual reached depth and the preset target depth, as shown in Figure 5. Depth errors were calculated from the deviations between each local minimum of the depth trajectory and the set target depth. This metric further revealed the differences in users’ depth perception errors between conditions with and without haptic feedback.
- **Success rate**: Success was quantified as a depth error within ±2500 micrometers. Figure 6 documents the number of successful and unsuccessful attempts made by the six participants to reach the set target depth within the experimental duration under both haptic and non-haptic feedback scenarios. This metric is critical for evaluating the effectiveness of micro-needle manipulation.
- **NASA-TLX scores**: To assess the cognitive and physical demands placed on participants, we used the NASA-TLX assessment. This tool evaluates mental (Q1), physical (Q2), and temporal (Q3) demands, as well as effort (Q4), performance (Q5), and frustration (Q6) experienced by participants while using the proposed framework. Scores range from 0 to 20 across six weighted subcategories, with lower scores indicating a lower workload or better task performance. This comprehensive assessment, shown in Figure 7, provides insights into the overall user experience and workload while using the microsurgical system.

#### 4.2.2. Results Analysis

We evaluated the effectiveness of haptic feedback based on the evaluation metrics mentioned above.

Table 5 shows that the mean depth errors of the six participants under the condition of haptic feedback are lower than those without haptic feedback, especially for the second and fourth participants. This indicates that the introduction of haptic feedback significantly reduces depth errors. The interquartile range (IQR) of the box plots in Figure 5 indicates that, without haptic feedback, the length of the box is much greater compared to when haptic feedback is present, particularly evident in participants two, four, and six. This suggests that the introduction of haptic feedback reduces the variability of depth errors, enhancing the reliability of the system. Furthermore, the symmetry of the depth error distribution appears to be enhanced with haptic feedback, suggesting that participants exhibit more consistent performance when tactile feedback is provided.

The first participant attempted to maneuver the micro-needle to the target depth three times without haptic feedback; unfortunately, all three attempts resulted in failure. With haptic feedback, the same participant completed only two attempts but achieved a 100% success rate. The second participant completed three tasks, both with and without tactile feedback, respectively. However, the outcomes were similar: this participant had zero success in completing tasks without haptic feedback. Conversely, under haptic feedback conditions, they completed three tasks successfully. The third participant achieved a 100% success rate both with and without haptic feedback. However, with haptic feedback, this participant completed one more task than without haptic feedback. The fourth participant completed four tasks successfully under haptic feedback conditions. However, without haptic feedback, only two tasks were completed, and out of these two attempts, only one was successful. The fifth participant completed five tasks under haptic feedback conditions, the highest among all participants. Out of the four attempts, the participant successfully achieved the target depth in four instances, with only one failure. In comparison, without haptic feedback, the fifth participant completed only three tasks with two failures. The sixth participant completed four tasks with haptic feedback and three tasks without it. Consequently, the participant experienced one failure in both scenarios: one failure out of four attempts with haptic feedback and one failure out of three attempts without haptic feedback.

The data analysis of the three evaluation metrics, depth, depth error, and success rate, indicates that haptic feedback not only improves the success rate of the task but also reduces depth error. The final evaluation metric is NASA-TLX scores. Figure 7 presents the participants’ ratings in mental demand, physical demand, temporal demand, performance, effort, and frustration questions under conditions with and without haptic feedback. A lower value indicates easier completion of the task due to lower demand. It is clear that, with haptic feedback, user ratings were notably lower compared to scenarios without haptic feedback, except for mental and physical demand scores, which showed no significant difference. Additionally, the scores for temporal demand with haptic feedback were nearly half of those without. Furthermore, in terms of frustration, the maximum score with haptic feedback was considerably lower than the minimum score without haptic feedback, indicating a significant difference. Lower scores suggest that the introduction of haptic feedback enhances participants’ performance in aspects of temporal demand, performance, effort, and frustration.

## 5. Discussion

User studies indicate that haptic gloves can enhance users’ perception of depth and reduce depth errors in microsurgical tasks. In defining the “success rate” as one of the evaluation metrics in our user study, we set a threshold of 2500 micrometers. The 2500-micrometer threshold was established based on user performance and served to assess performance disparities between participants with and without haptic feedback; it did not serve as a parameter for evaluating controller tracking accuracy. In user tracking experiments, users were asked to move their hands within the optimal range from the camera to maintain high accuracy. When the hands move out of this optimal range, tracking accuracy will be compromised. Additionally, the article solely demonstrates the on/off functionality of the haptic interface through inflation and deflation, aiming to showcase that our haptic interface is entirely soft, lightweight, and cost-effective. However, it is important to note that it is customizable for different tasks, and its potential for shape rendering remains to be developed. Finally, the integration of haptic feedback enhances users’ performance in terms of temporal demand, performance, effort, and frustration. However, it does not result in significant improvements in mental and physical demand, likely due to the increased cognitive burden.

## 6. Conclusions and Future Work

To conclude, in this paper, our team successfully designed and constructed a lightweight, affordable, and wearable handheld haptic controller tailored for RAMS applications. More specifically, we developed a soft haptic interface that can be easily customized and fabricated on a wearable glove-like wearable controller. A key innovation in our design process was the use of liquid and solid hybrid 3D co-printing technology for fabricating pneumatically actuated haptic arrays. This approach enables the direct printing of liquid within micro/meso fluidic channels, significantly streamlining the fabrication process by reducing the need for extensive post-processing. The liquid support ensures both structural stability during printing and facilitates easy removal afterward. Moreover, the motion tracking system designed for RAMS employs a multi-sensor fusion approach, combining the advantages of two vision-based tracking methods and IMU sensor data.

Our experiments involved a comparative analysis of three different filters, where in the UKF emerged as the most accurate and reliable option for sensor fusion during motion tracking. We validated the effectiveness of haptic feedback through user studies focused on a needle insertion task. Results demonstrated that the performance of participants improved significantly thanks to the haptic feedback. Moreover, as motion tracking technology advances, there is the potential to integrate higher-precision tracking methods into our RAMS. Further comparative testing against other controllers through user studies will be conducted before determining the clinical potential of the proposed wearable haptic controller.

We provide users with a sense of depth through haptic interfaces to enhance their perception of depth. A key focus of our future work is to explore the capabilities of shape rendering in haptic interfaces.

## Figures and Tables

**Table 1:** The time (seconds) for haptic interfaces to revert to their initial state after venting for 0.5, 1, and 1.5 s, utilizing a 0.3 mm printing thickness, across materials with three different Shore A hardness levels (30 A, 50 A, and 70 A).

Shore A	30 A	50 A	70 A
0.5 S	0.22	0.20	0.19
1 S	0.35	0.31	0.26
1.5 S	0.45	0.31	0.30
Mean	0.34	0.27	0.25

**Table 2:** The time (seconds) for haptic interfaces to revert to their initial state after venting for 0.5, 1, and 1.5 s, utilizing a 0.2 mm printing thickness, across materials with three different Shore A hardness levels (30 A, 50 A, and 70 A).

Shore A	30 A	50 A	70 A
0.5 S	0.21	0.20	0.17
1 S	0.35	0.38	0.27
1.5 S	0.42	0.32	0.30
Mean	0.32	0.27	0.25

**Table 3:** Detection rates for pose estimation methods at different angles.

Pose Estimation Method	Depth (cm)	${\bar{n}}_{i}$	$\bar{N}$	$\bar{\gamma }$
Marker-less	4	157	194	0.81
Marker-based	4	281	283	0.99
Marker-less	8	184	207	0.89
Marker-based	8	246	253	0.97
Marker-less	12	119	172	0.69
Marker-based	12	149	259	0.58

**Table 4:** Comparison of RMSE and Total path length *D* errors for KF, EKF, and UKF filter across different planes (unit: cm).

Filter	Plane	RMSE	*D* Errors
KF	*x*−*y*	0.306	0.848
EKF	*x*−*y*	0.224	0.276
UKF	*x*−*y*	0.246	0.152
KF	*x*−*z*	0.642	2.426
EKF	*x*−*z*	0.640	2.842
UKF	*x*−*z*	0.580	2.722
KF	*y*−*z*	0.730	2.418
EKF	*y*−*z*	0.616	2.648
UKF	*y*−*z*	0.559	2.672

**Table 5:** Mean depth error (μm). Depth error signifies the variance between the local depth minima in the trajectory and the predetermined target depth. The figure illustrates the mean error associated for every user, considering both scenarios, with and without haptic feedback.

	User1	User2	User3	User4	User5	User6
with	1525.7	436.48	728.3	373.8	1489.4	1422.3
without	3988.3	7349.4	837.5	5464.5	2347.7	2746.7

## Data Availability

The data presented in this study are available on request from the corresponding author.
